# Individualized Treatment of Neovascular Age-Related Macular Degeneration: What are Patients Gaining? Or Losing?

**DOI:** 10.3390/jcm4051079

**Published:** 2015-05-21

**Authors:** Michael W. Stewart

**Affiliations:** Department of Ophthalmology, Mayo Clinic Florida, 4500 San Pablo Rd., Jacksonville, FL 32224, USA; E-Mail: stewart.michael@mayo.edu; Tel.: +1-904-953-2232; Fax: +1-904-953-7040

**Keywords:** age-related macular degeneration, as-needed therapy, bevacizumab, choroidal neovascularization, monthly therapy, ranibizumab, treat and extend

## Abstract

The widespread use of drugs that bind diffusible vascular endothelial growth factor (VEGF) has revolutionized the treatment of neovascular age-related macular degeneration (AMD). The pivotal ranibizumab and aflibercept registration trials featured monthly intravitreal injections for 12 months, during which visual acuities and macular edema rapidly improved for the first 3 months and modest gains or stabilization continued until the primary endpoint. In many subsequent trials, patients were evaluated monthly and treated as-needed (PRN) according to the results of visual acuity (VA) testing, fundus examinations and optical coherence tomography scans. Compared to monthly-treated control groups, PRN treated patients require fewer injections during the first year but they also experience smaller VA gains (1–3 letters). A small number of prospective trials that directly compared monthly with PRN therapy showed that VA gains with discontinuous therapy lag slightly behind those achieved with monthly injections. Physicians recognize that monthly office visits with frequent intraocular injections challenge patients’ compliance, accrue high drug and professional service costs, and clog office schedules with frequently returning patients. To decrease the numbers of both office visits and anti-VEGF injections without sacrificing VA gains, physicians have embraced the treat-and-extend strategy. Treat-and-extend has not been studied as rigorously as PRN but it has become popular among both vitreoretinal specialists and patients. Despite the possible risks associated with discontinuous therapy (decreased VA and increased macular fluid), most physicians individualize treatment (PRN or treat-and-extend) for the majority of their patients. This review chapter explores the many advantages of individualized therapy, while balancing these against suboptimal responses due to the decreased frequency of anti-VEGF injections.

## 1. Introduction

Age-related macular degeneration (AMD) represents the leading cause of blindness in industrialized countries [1]. Ninety percent of AMD patients suffer from the non-neovascular or “dry” type, which is characterized by drusen and retinal pigment epithelium (RPE) mottling, clumping, and atrophy. Since most of these patients experience only mild decreases in visual acuity (VA) or metamorphopsia, dry AMD causes only 10% of AMD-related blindness [2].

Most AMD-related blindness results from the neovascular or “wet” form of AMD [3], which is characterized by neovascular growth from the choroid into the sub-RPE or subretinal spaces. Prevalence rates for neovascular AMD are high across both geographical boundaries and racial divides. The Blue Mountains Eye Study found a 10-year neovascular AMD incidence of 2.2% for patients past the age of 49 years, 2.0% for those from 60–69 years, and 12.4% for those 80 years and older [4]. Similar average incidence rates were found in a comprehensive study of the Icelandic population [5].

The etiology of AMD appears complex and remains incompletely understood despite a large body of experimental data and clinical trial findings. Age-related macular degeneration results from both predisposing genetic abnormalities and environmental factors. Current evidence suggests that AMD results from a lifetime of metabolic insult to the photoreceptors and RPE. For unknown reasons, some patients accumulate a thick lipoprotein multi-layer external to the RPE basal lamina, which has been referred to as “The oil spill in Bruch’s membrane” [6]. Some of these pro-inflammatory molecules contribute to local oxidative stress and upregulate several chemokines and cytokines, with vascular endothelial growth factor (VEGF) being one of the most important.

Vascular permeability factor (VPF) was discovered in 1983 [7] but its primary structure could not be characterized with the available technology. Ferrara [8] and Thompson [9] independently discovered VEGF in 1989, and the newly developed protein sequencing technology showed that this was the previously reported VPF. Subsequent research has identified several VEGF molecules that segregate into 7 families [10]. Four VEGF-blocking drugs have been approved by the United States Food and Drug Administration (US FDA) for the treatment of ocular neovascular conditions and advanced carcinomas.

Progressive exudation, bleeding, and fibrosis characterize the natural history of neovascular AMD with 40% of untreated eyes losing at least 30 letters of vision [11]. The recently developed anti-VEGF drugs represent the first treatments that prevent moderate and severe vision loss in nearly all neovascular AMD patients and improve VA in the majority. Level I evidence now supports the use of pharmacologic VEGF blockade in patients with neovascular AMD [12,13,14], diabetic macular edema (DME) [15,16,17,18], and macular edema due to central and branch retinal vein occlusions [19,20,21,22,23]. The phase III neovascular AMD trials were completed first so much of our pharmacokinetic, pharmacodynamic, and safety data come from the AMD population. Not surprisingly, the majority of anti-VEGF doses have been administered to patients with neovascular AMD. As the large baby boomer populations within industrialized nations approach 70 years of age, the number of anti-VEGF injections is expected to increase significantly over the next 20 years.

Some patients with neovascular AMD require long, sometimes unending series of anti-VEGF injections, whereas fluorescein angiography demonstrates that choroidal neovascularization may permanently regress in other patients after only a small number of injections. Administering monthly injections to this latter group incurs high direct and indirect costs, and requires significant time commitments by both patients and their caretakers [24], most of which would be unnecessary. To prevent over-treating some patients and to control both costs and risks, the majority of treating physicians attempt to individualize therapy.

Compared to a regimen of monthly injections, individualized therapy usually decreases the number of injections and the number of patient visits to the physician’s office. These individualized regimens result in clear advantages in cost and time commitment [25] but substantial data suggests that visual outcomes may also suffer [26,27]. This chapter will explore both the advantages and disadvantages of individualized therapy for AMD to provide physicians a framework with which they may choose the best therapy for each patient.

## 2. History

The phase III MARINA trial [13] demonstrated that monthly injections of ranibizumab (Lucentis^®^, Genentech, S. San Francisco, CA/Roche, Basel, Switzerland) improve mean VA in patients with occult subfoveal choroidal neovascularization (CNVM) due to AMD. Even though no effective alternative therapy existed at that time, 14% of patients were unable to attend monthly visits and complete the 2-year trial. The authors believed that a regimen of monthly intravitreal anti-VEGF injections, though effective, was difficult to sustain [13]. Other authors opined that monthly injections are impractical for many patients because of the associated physical and psychological burdens, and the risks of adverse ocular events such as endophthalmitis [28].

Several treatment frequencies and strategies have subsequently been studied to improve patient compliance and decrease the risks of adverse events and the costs of therapy. Following the pivotal phase III, monthly-injection registration trials MARINA and ANCHOR [13,14], several trials (PIER, EXCITE, SUSTAIN, HORIZON, and CATT, IVAN) explored less frequent therapy [26,27,29,30,31,32]. Results of these trials vary but VA improvements generally lag behind those achieved in MARINA and ANCHOR. Comparing results from different trials must be done with great care, however, because each trial uses different entry criteria and retreatment thresholds.

Due to the successes of MARINA and ANCHOR, ranibizumab accounted for 10% of Medicare Part B drug expenses in 2010 [33]. The high cost of treatment was one of the major reasons for the CATT trial and is a factor in shaping both the DRCR.net Protocol T for diabetic macular edema [34] and the SCORE 2 trial for edema due to central retinal vein occlusion [35]. Because regimens using the increasingly popular ranibizumab ($1950/dose) and aflibercept ($1850/dose) are expensive, particularly compared to those with bevacizumab ($50–$80/dose), the cost of anti-VEGF treatment has emerged as a powerful driver of individualized therapy.

## 3. Present Data

### 3.1. General

To predict the durations of clinical action achievable with intravitreal anti-VEGF drugs, some authors have used biochemical, physiologic, and clinical data to create mathematical models. A model based on VEGF-binding affinities, expected time-dependent drug concentrations within the eye (based upon the intravitreal half-lives from monkey experiments), and successful results from the ANCHOR and MARINA trials, was developed to predict the relative durations of action of intravitreal bevacizumab and ranibizumab. A 27-day to 38-day duration of action for bevacizumab (compared to 30 days for ranibizumab) was predicted [36]. Using the same methodology, the clinical effects of aflibercept (Eylea^®^, Regeneron, Tarrytown, NY) were predicted to last approximately 80 days (compared to 30 days for ranibizumab) [37]. The model correctly predicted the extended action of aflibercept (nearly 3 months in year 2 of the VIEW trials) but underestimated the duration of ranibizumab [38].Responses to anti-VEGF therapy vary among individuals and may be influenced by genetic factors, stimuli for VEGF expression, clearance of endogenous VEGF, expression of other angiogenic and anti-angiogenic factors, diet and environmental factors, duration of disease, and composition of the neovascular lesions [39]. Van Asten *et al* evaluated the clinical responses of 391 patients, 47 of whom were non-responsive to ranibizumab. They derived a clinical prediction rule from several independent factors: age, baseline visual acuity, diabetes mellitus and accumulation of high risk alleles (complement factor H, ARMS2, and VEGF-A genes) [40].

Visual acuity improvements with monthly anti-VEGF therapy generally follow a bi-modal pattern with rapid increases during the first 3 months followed by improvements of less than 2 letters for the balance of the first year. Most clinical trials, therefore, begin with 3 monthly injections followed by either continued monthly therapy [13,14] or a switch to individualized therapy [26,27].

Monthly treatment increases the global cost of therapy, floods physicians’ offices with returning patients, and limits access to care for patients with new medical and surgical conditions. Not surprisingly, physicians have adopted individualized strategies to decrease these burdens. In the 2008 American Society of Retina Specialists Preferences and Trends survey, physicians reported using individualized treatment regimens on 88% of patients. Physicians understand that 10% to 20% of patients in PRN trials require fewer than 4 injections the first year, so committing to monthly injections forfeits the opportunity to identify them.

Poorly informed patients become more susceptible to external influences, so physicians play an important role in influencing many patients’ decisions about the care they receive [41]. Individualized care requires shared decision making between the patient and doctor, but the demographics of the AMD population often makes this process more difficult because older patients may be less receptive to receiving information and advice [42,43]. Nevertheless, detailed discussions between physicians and patients need to be undertaken before embarking on treatment regimens that incorporate individualized therapy.

### 3.2. Clinical: Extended Interval Therapy

MARINA and ANCHOR showed that monthly ranibizumab injections improved vision in most eyes with neovascular AMD but they did not attempt to identify eyes that required more or less frequent dosing. One of the first trials to explore extended-interval dosing was the phase IIIb PIER trial [29]. One hundred eighty-four patients were randomized to receive sham injections or ranibizumab (0.3 mg or 0.5 mg). Patients received 3 monthly injections followed by quarterly injections. Mean VA changes (in letters) from baseline to 1 year were −16.3 (sham), −1.6 (0.3 mg ranibizumab), and −0.2 (0.5 mg ranibizumab). By month 12, ranibizumab treated patients lost an average of −4.5 letters from their peak VA at month 3 despite arrested CNVM growth on fluorescein angiography and decreased leakage. On the other hand, 40% of patients maintained vision gains achieved during the first 3 months. Exploratory analysis of the PIER data showed that eyes could be stratified based on gains achieved during the first 3 months; patients who maintained vision after the first 3 months were most likely to maintain vision after the month 11 injection [44]. After review of MARINA, ANCHOR, and 12-month PIER data, the sponsors believed that all PIER patients should be eligible to receive ranibizumab during the second year. As a result, sham treated patients were crossed over to receive 0.5 mg ranibizumab quarterly and a second protocol amendment (rollover) subsequently allowed all patients to receive 0.5 mg ranibizumab monthly. Sham patients who crossed over and then rolled over lost −3.5 letters after rollover. Patients originally receiving 0.3 mg and 0.5 mg ranibizumab gained an additional +2.2 and +4.1 letters after rollover to monthly injections. The authors estimated that the recovery of vision with monthly injections initiated after an initial 5-line loss due to infrequent injections may be the same as with the initial loading doses [29,45].

Based on data from the MARINA, ANCHOR and PIER trials [13,14,29], Holz and colleagues developed a patient-specific model of disease progression, with emphasis on VA changes. Their simulated monthly and quarterly treatment models closely resembled results from the clinical trials. The model predicted an average of 5.1 ranibizumab injections during the 9 months after administration of the 3 induction doses [39].

Many physicians worry that decreasing the frequency of injections, as was done in PIER, may allow the CNVM to reactivate and increase the risk of hemorrhages and permanent loss of vision. Monthly ranibizumab injections have been associated with fewer retinal hemorrhages than sham or photodynamic therapy (PDT), but quarterly injections of ranibizumab (PIER) were associated with the same number of hemorrhages as sham injections. Additionally, there do not appear to be differences in hemorrhages among patients taking aspirin, clopidogrel, or warfarin [46].

The 12-month EXCITE trial [30] was similar to PIER except that it also had a monthly control group. Three hundred fifty-three patients were randomized 1:1:1 to receive 0.3 mg or 0.5 mg ranibizumab quarterly or 0.3 mg ranibizumab monthly. All patients received a loading dose of 3 consecutive monthly injections followed by a 9-month maintenance phase. The average VA improved by +4.9 (0.3 mg ranibizumab quarterly), +3.8 (0.5 mg ranibizumab quarterly), and +8.3 letters (0.3 mg ranibizumab monthly), and average improvements in macular thickness were −96 µm, −105 µm, and −105 µm. Between months 3 and 12, quarterly treated patients lost an average of −1.8 and −2.8 letters and monthly treated patients gained an average of +0.8 letters. The first noticeable differences in VA among the groups were at month 4 (2 months after the last loading dose) suggesting that the duration of ranibizumab was less than 2 months for many patients. The proportions of patients who lost <15 letters were 93.3%, 91.5%, and 94.8% and who gained >15 letters were 14.2%, 17.8%, 28.7%. The study failed to meet the pre-determined non-inferiority margin of 5 letters. More patients in the most intensely treated group (0.3 mg monthly) discontinued ranibizumab due to adverse events. A sub-group analysis showed that 41.6% of quarterly treated patients maintained vision after the loading phase [30].

A 48-week trial with quarterly bevacizumab (after 3 loading injections every 6 weeks) resulted in +10.6 letters of visual improvement and significant macular thinning (CST: 343 µm → 222 µm) through 12 weeks. When the injection frequency was decreased to quarterly, however, the improvement in VA from baseline dropped to +3.9 letters and the macular thickness increased to 268 µm [47].

### 3.3. Clinical—PRN

The phase I and II ranibizumab trials showed considerable variability in duration of drug action among patients [48], suggesting that some eyes required monthly injections while others could be treated much less frequently. One of the first investigator-initiated, individualized therapy trials was PrONTO [49,50], which featured monthly examinations and PRN ranibizumab injections. The sponsor mandated that patients receive 3 monthly loading injections of ranibizumab because most ANCHOR and MARINA patients continued to improve through 3 months. Subsequent injections in PrONTO were administered PRN, based on findings from the monthly examinations. Retreatment requirements for PrONTO were strict, patients were followed closely, and the retention rate was high. Retreatment was required for eyes that developed a 5-letter VA loss associated with fluid, increase in CRT of 100 µm, new onset of classic CNVM, new hemorrhage, or persistent fluid after the last injection [49]. The mean 1-year VA improvement was +9.3 letters, with 35% of patients improving by at least 3 lines. Patients received a mean of 9.9 injections over 24 months but there was considerable inter-patient variability (range: 3 to 25 injections) [50]. PrONTO produced VA improvements comparable to those achieved in ANCHOR and MARINA but with 59% fewer injections. PrONTO showed that VA measurements and clinical examinations remain important for assessing disease activity, but since VA may vary between visits and may not be reflective of AMD disease activity [51] they remain insufficient predictors. PrONTO demonstrated that changes in OCT may precede visual deterioration, thereby establishing the importance of OCT in guiding individualized therapy [49,51]. Finally, since PrONTO was a small trial (40 patients) without a control group, we don’t know if these eyes could have done even better had they been given monthly injections.

The first prospective comparison of ranibizumab to bevacizumab was a 6-month, randomized PRN trial [52]. Patients were treated monthly × 3 and followed monthly with OCT-guided reinjections as needed. The average visual acuities peaked at 3 months, and at 6 months they remained improved in both groups (bevacizumab from 31.6 letters to 46.4 letters; ranibizumab from 30.4 Letters to 37.4 letters; *p* = 0.09). The average numbers of required injections were 5 (bevacizumab) and 4 (ranibizumab). The average improvements in macular thickness differed between groups (bevacizumab: −35 µm, ranibizumab: −102 µm) [52].

The phase IIIb SAILOR trial [53] evaluated the safety of ranibizumab in 490 treatment-naïve subjects. Patients received 3 monthly injections followed by as-needed therapy based on OCT and VA criteria. In contrast to PrONTO, SAILOR did not include new hemorrhage or new classic CNVM with persistent fluid after the last injection in the retreatment criteria. Furthermore, the frequency of mandated office visits during the first year decreased from monthly to quarterly after the induction period. Average visual acuities improved by +5.8 to +7.0 letters after 3 injections but had decreased to +0.5 to +2.3 letters at month 12. VA improvement of >15 letters was achieved by 19.2% of patients [53]. Compared to PrONTO, poorer VA results in SAILOR may be explained by less frequent follow-ups and less stringent retreatment criteria.

The EXTEND-I [54] was the first study to demonstrate the safety and efficacy of ranibizumab in the Japanese population. Patients in this phase I/II, open label study were treated with either a single injection (Group A) to assess drug safety or multiple injections (Group B) of ranibizumab (0.3 mg or 0.5 mg) to assess efficacy. Mean VA in Group B improved by an average of +9.5 to +10.5 letters at 12 months, and 31% to 37% of patients improved by >15 letters. Blood was sampled at various times (up to 14 days) after intravitreal injections to determine pharmacokinetic parameters. Serum elimination half-lives—t_½_ was 6.56 days for the 0.3 mg dose and 7.85 days for 0.5 mg—reflected slow drug movement from the eye into the serum [28]. Treatment past month 12 was offered only to patients in Group B. Seventy patients were treated PRN with ranibizumab (0.3 mg or 0.5 mg) according to BCVA (when vision decreased by ≥5 letters at 2 consecutive visits) and other parameters. The mean numbers of ranibizumab injections were 4.19 and 4.27 per year, and BCVA changes were −3.6 and −2.2 letters at 35 months [54]. The results achieved with the PRN regimen between months 12 and 35 suggest that many patients do not require monthly injections to maintain initial gains from monthly therapy.

A retrospective chart review of ranibizumab treated patients, either as 3 injections followed by PRN, or PRN from baseline, found that the mean numbers of injections at 6 months were 6.0 and 4.5 with similar mean changes in VA (+4.2 letters) and thickness. More patients in the loading dose group (29.8% *vs.* 12.9%) improved by +15 letters. The authors claim that optimal VA is achieved with 3 monthly loading doses followed by a greater frequency of injections [55]. Results from this study do not mimic PrONTO [49,50], but they more closely resemble those from SUSTAIN (+3.6 letters, 5.7 injections) [31] and the monotherapy arm of MONT BLANC (+4.4 letters, 5.1 injections) [56].

The CATT was the first and largest of the national ranibizumab *versus* bevacizumab trials [26,57]. CATT randomized 1208 patients to receive bevacizumab or ranibizumab, monthly or discontinuously (monthly assessments with PRN treatment). At 12 months, the average improvements in VA were +8.0 letters (bevacizumab monthly), +8.5 letters (ranibizumab monthly), +5.9 letters (bevacizumab PRN), and +6.8 letters (ranibizumab PRN). No significant differences in VA were noted between drugs, but the VA comparison between bevacizumab monthly and PRN was inconclusive. The greatest decrease in CRT occurred in the ranibizumab monthly group. The mean numbers of PRN injections were 6.9 (ranibizumab) and 7.7 (bevacizumab). At one year, the mean lesion sizes were unchanged in patients receiving monthly injections but were slightly larger in patients receiving PRN injections (*p* = 0.047), and leakage on fluorescein angiography was greater in patients receiving PRN injections. The development of systemic adverse events (SAEs) was greater with bevacizumab than ranibizumab (1.29×; 95% confidence intervals: 1.01–1.66) but most of these were not for problems usually associated with VEGF. The proportions of patients experiencing atherosclerotic events (2%–3%) were similar among all groups (*p* = 0.97).

Patients enrolled in CATT had an older average age and better baseline VA compared to those in most other AMD trials. Nonetheless, VA gains with PRN ranibizumab were the best of any large, multi-center individualized trial. This may have been due to rigorous retreatment guidelines because any fluid (not just thickening) demanded retreatment [57]. Despite this aggressive retreatment strategy, the proportions of patients with no fluid on OCT at 52 weeks remained significant: bevacizumab PRN—19%; ranibizumab PRN—24%; bevacizumab monthly—26%; ranibizumab monthly—43%. CATT uncovered 3 differences between the drugs: (1) ranibizumab was better at drying the retina; (2) VA results with ranibizumab tended to be better; (3) ranibizumab may last longer (according to results from the PRN arms) [58].

Patients who had been treated monthly during year 1 were randomized to receive either monthly or as-needed injections during year 2. The mean changes in VA with each drug were similar between years 1 and 2 (bevacizumab − ranibizumab = −1.4 letters; *p* = 0.21) but the gains in VA were greater with monthly than as-needed therapy (+2.4 letters; *p* = 0.046). Switching from monthly to as-needed injections during year 2 resulted in a −2.2 letter loss (*p* = 0.03) and a lower proportion of eyes without fluid (19%; *p* < 0.001). The mean total retinal thickness changed little in patients treated monthly but increased in patients that were switched to PRN (*p* = 0.004). The proportions of eyes without fluid ranged from 45% with monthly ranibizumab down to 13.9% with as-needed bevacizumab (drug: *p* = 0.0003; regimen: *p* < 0.0001). Not surprisingly, the average macular thickness of patients receiving monthly therapy was 29 µm less than those receiving PRN therapy. More geographic atrophy was seen in eyes receiving monthly injections (*p* = 0.007). Ten of 11 endophthalmitis cases occurred in monthly treated groups. The proportions of patients experiencing arteriosclerotic events were similar for patients treated with each drug. The rates of death were similar with both drugs and the proportion of eyes with 1 or more serious adverse events was higher with bevacizumab than ranibizumab (39% *vs*. 31.7%, *p* = 0.009). Over the 2-year study, the incidences of death, stroke, and myocardial infarction did not differ between drugs but the incidence of serious adverse events was higher in the bevacizumab group (risk ratio = 1.3) [26].

The IVAN trial (United Kingdom) [27,59] randomized 610 patients to receive monthly or discontinuous ranibizumab or bevacizumab. Unlike CATT which required a minimum of 1 initial injection and 1 with each retreatment, IVAN mandated 3 monthly injections initially and with each retreatment. For patients receiving monthly injections, those treated with bevacizumab gained −1.99 fewer letters than those receiving ranibizumab. Visual improvements in patients receiving discontinuous therapy were nearly the same as in those receiving continuous therapy (discontinuous − continuous = −0.35 letters). Patients in the discontinuous groups received an average of 7 injections by month 12. Total foveal thickness was 9% less in patients receiving continuous therapy but did not differ by drug. Fewer patients receiving bevacizumab had thromboembolic events or heart failure. Patients receiving bevacizumab had lower systemic VEGF concentrations, with slightly higher levels in patients receiving discontinuous treatments [59].

At 2 years the average difference in VA between eyes receiving bevacizumab and those receiving ranibizumab was −1.37 letters and between discontinuous and continuous therapy was −1.63 letters. Macular thickness was similar by drug but CST was 9% less in eyes receiving continuous treatment. The odds ratio of developing new geographic atrophy was 1.47 for continuous *versus* discontinuous therapy (*p* = 0.033). Overall safety profiles did not differ by drug, but mortality was lower with continuous therapy (odds ratio of 0.47; *p* = 0.05) [27].

SUSTAIN [31] was a 12-month, phase III, open-label study of 512 treatment-naïve patients. Patients received monthly ranibizumab 0.3 mg × 3 followed by PRN injections if the vision worsened by >5 letters or the CRT increased by >100 µm. Average VA improved by +5.8 letters at month 3 and +3.6 letters at month 12. After the 3 initial doses, patients in SUSTAIN required 70% fewer injections—an average of 2.7 during the final 9 months—than did patients in ANCHOR but with 80% of the treatment effect. The 1-year VA results were disappointing and, in hindsight, the authors stated that a 50 µm threshold for retreatment would probably have been better than 100 µm [31].

A combination therapy trial compared 3 loading doses of ranibizumab followed by PRN treatment, *versus* ranibizumab together with photodynamic therapy (PDT). Eyes with classic and occult CNVM were included. All eyes lost fewer than 15 letters except for one from the combination therapy arm. Mean gains in VA were greater in eyes receiving monotherapy (+12 letters *vs*. +3.2 letters) though CRT improvement was the same in both groups. Combination therapy decreased treatment burden, as both the time to retreatment after induction was longer (*p* = 0.002) and fewer retreatments were required by the combination group (*p* = 0.015) [60].

Other trials comparing ranibizumab with PDT produced similar results. The RADICAL trial [61] showed that half-fluence PDT combined with ranibizumab and dexamethasone produced similar 1-year gains in VA as ranibizumab monotherapy, but with fewer injections. The MONT BLANC trial [56] produced gains of +4.4 (ranibizumab monotherapy) *vs*. +2.5 (PDT + ranibizumab) letters but there was no difference in the treatment-free intervals. The failure of PDT/ranibizumab combination therapy to match VA gains produced by ranibizumab monotherapy may result from PDT-induced choroidal hypoperfusion [56]. Unfortunately, anti-VEGF injections do not prevent PDT-produced blood-barrier breakdown (BBB), which is why some investigators advocate triple therapy by adding a corticosteroid such as dexamethasone [62]. Though proponents claim that this leads to excellent VA gains with fewer injections, controlled trials have not been performed.

A Canadian group [63] retrospectively studied 192 eyes treated PRN with ranibizumab (50) or bevacizumab (142). Three monthly injections were followed by PRN injections, with retreatment based on findings from OCT, angiography, and clinical examinations. Patients received an average of 4.92 (ranibizumab) and 4.75 (bevacizumab) injections during the first year. Average VA improved from 0.69 LogMAR to 0.55 LogMAR (ranibizumab) and 0.70 LogMAR to 0.67 LogMAR (bevacizumab). After the initial 3 injections, 20% of ranibizumab treated eyes and 26% of bevacizumab eyes needed no additional injections [63].

HORIZON [32] was an open-label extension trial for patients from 3 randomized control trials (MARINA [13], ANCHOR [14], and FOCUS) [64], each of which had followed patients monthly for 24 months (853 of 1064 eligible patients). At the time of their enrollment into HORIZON, ranibizumab treated patients had already improved by +4.6 to +10.7 letters, compared to losses of −7.8 to −14.9 letters in the control groups. HORIZON patients had been initially treated with monthly ranibizumab (600), control and subsequent cross-over to ranibizumab from PDT or sham (190), or were untreated (63). In HORIZON, ranibizumab was administered at the investigator’s discretion with no pre-specified conditions. Follow-up visits were originally required every 6 months, though this was subsequently changed to every 3 months. Primary outcome measures were incidences of ocular and non-ocular adverse events. Approximately 30% of patients never received ranibizumab injections in HORIZON, a figure that did not differ between patients initially treated with ranibizumab or those that had been untreated. The majority of patients receiving ranibizumab injections were injected within the first 6 months and the cumulative average numbers of injections by 1, 2, and 3 years were 2.2, 4.2 and 4.3. At 2 years into HORIZON the mean changes in VA from the initial baselines in MARINA, ANCHOR, and FOCUS were +2.0 letters for patients initially treated with ranibizumab *versus* −11.8 letters for those who were untreated or crossed over. Average VA changes during HORIZON were −9.1 letters in patients initially treated with ranibizumab *versus* −6.5 letters for patients who were cross-over and untreated. Only 1 mild case of endophthalmitis occurred in 3552 injections. The proportions of patients with cataracts were lower in patients originally untreated (6.3%) *versus* those treated with ranibizumab (12.5%) or crossed over (12.1%). The study was run at a time when the importance of strict, aggressive retreatment criteria and short follow-up intervals for patients with AMD had not yet been established. The authors believed that transition from monthly to PRN injections with less frequent monitoring led to destabilization of the AMD [32].

MANTA [65] was a national (Austria) ranibizumab/bevacizumab trial that randomized 317 patients to receive 3 monthly injections of ranibizumab or bevacizumab followed by monthly evaluations and treatment as-needed. Visual acuities improved quickly, peaked at 3–4 months, and then decreased by an average of −2 letters through 12 months. Average improvements in VA at 12 months were +4.9 letters (bevacizumab) and +4.1 letters (ranibizumab). Mean CRT decreased from 374 µm to 288 µm in patients receiving bevacizumab and from 365 µm to 275 µm in patients treated with ranibizumab. The average numbers of re-treatments were 6.1 (bevacizumab) and 5.8 (ranibizumab) [65].

GEFAL [66] was a national (France) ranibizumab/bevacizumab trial that randomized 501 patients to receive 3 monthly bevacizumab or ranibizumab injections followed by monthly examinations and as-needed injections for 1 year. The mean numbers of injections were 6.8 (bevacizumab) and 6.5 (ranibizumab). Eyes receiving bevacizumab improved by +1.8 letters more than those receiving ranibizumab (+5.4 *vs.* +3.6). The overall improvements in VA were inferior to those seen in CATT, perhaps because a larger proportion of patients were enrolled with very poor VA (<37 letters). CMT was reduced by 95 µm (bevacizumab) and 107 µm (ranibizumab) [66].

Unfortunately, none of the subsequent as-needed studies have been able to reproduce the VA results achieved in PrONTO. The national French (GEFAL) [66] and Austrian (MANTA) [65] trials showed 1-year VA improvements of +3.6 to +5.4 letters without a demonstrable difference between bevacizumab and ranibizumab treated groups. CATT [26] and IVAN [27] randomized patients to both drug (bevacizumab or ranibizumab) and regimen (monthly or discontinuous) and patients treated as-needed with either ranibizumab or bevacizumab gained fewer letters of vision (−0.35 to −2.1 depending on the treatment group and trial) than did those receiving monthly injections. A *post hoc* analysis of both studies showed that there was a significant difference in visual acuity gains according to treatment regimen—less improvement with discontinuous therapy—but not according to drug selection.

CATT [26] showed that patients receiving discontinuous therapy had slightly higher incidences of systemic adverse events compared to those treated with monthly injections. These findings are counter-intuitive and the reasons for them are unclear; validation will have to await the publication of larger prospective trials. For ocular adverse events such as endophthalmitis that occur due to the injection procedure itself, patients receiving as-needed and T&E injections should enjoy lower risks compared to those receiving monthly injections.

The CATT [26] and IVAN [27] trials found an increased incidence of geographic atrophy (GA) of the RPE in patients receiving monthly as opposed to discontinuous anti-VEGF injections, but there were no significant differences between patients treated with ranibizumab *versus* bevacizumab. The reason for advancing GA in these eyes is not known but it may be due to constantly depressed VEGF concentrations at the level of the RPE and choriocapillaris, or localized damage to the RPE due to the CNVM [67].

Monthly anti-VEGF therapy produces superior VA results compared to discontinuous therapy through 2 years but long term follow-up of patients from the early ranibizumab registration trials shows that average VA steadily worsens and by 7 years returns to baseline [68]. The primary reason for late VA loss in these eyes is progressive GA. If the results of CATT and IVAN are accurate then physicians may consider limiting anti-VEGF injections in eyes that may be expected to require long-term therapy. Patients might give up a couple of letters of VA in the short-term (2 years) but may be rewarded by less GA in the long-term. Data supporting this contention, however, are not available and additional studies that modify injection frequency in eyes with GA are needed to support this.

### 3.4. Clinical—Treat and Extend

As-needed treatment of neovascular AMD has been extensively studied in both retrospective reviews and prospective trials but it falls short of being the ideal strategy. Fewer intravitreal injections are required but the number of clinic visits remains high. Six-week as-needed intervals have been attempted but neovascularization reactivates within 6 weeks in too many patients. To decrease both the numbers of injections and clinic visits, and to maintain a fluid-free macula for as long as possible, the treat-and-extend strategy (T&E) was devised. This strategy begins with monthly loading injection(s)—generally 1 to 3—after which the patient returns in 4 weeks and is evaluated for signs of disease activity. The patient receives an injection and if the disease is active, returns in 4 weeks for another; if no evidence of disease activity is seen, the patient returns in 5 or 6 weeks. The patient receives an injection at each visit with the results of the examination determining the interval to the next visit. If inactive, the interval is extended by 1 or 2 weeks; if active, the interval is decreased by 2 weeks and then kept constant. Generally, once the optimal treatment interval is determined for a given eye, it usually remains constant.

In one of the first treat-and-extend reports [69], 18 eyes of 16 patients were treated for at least 24 months. Patients received 3 monthly injections and were then extended by 2-week intervals when they were dry. Average LogMAR VA improved from 0.53 to 0.41 at 3 months and to 0.52 at 24 months. Eighty-three percent of eyes had edema throughout the study and one eye developed geographic atrophy. The maximum interval was limited to 10 weeks because the investigators were concerned that a devastating hemorrhage could occur due to withdrawal of VEGF suppression. Fortunately, no sight-threatening hemorrhages occurred in this study [69].

In another study [70], 92 treatment-naïve eyes received monthly injections of ranibizumab until dry, with treatment intervals then extended by 2 weeks. Patients whose interval was extended to 12 weeks were given the option of continued treatment at 12-week intervals or no treatment with a subsequent return in 8 weeks. Mean VA improved from 20/135 to 20/77 at 1 year and 20/83 at 2 years. Ninety-six percent of patients lost fewer than 3 lines of VA and 32% gained 3 lines. The mean numbers of injections during the first and second years were 8.36 and 7.45 and the mean maximum extended period was 79.9 days [70].

A retrospective study compared patients treated with either treat-and-extend or as-needed [71]. Better VA improvement was seen in the T&E group at 1 year (+10.8 *vs.* +2.3 letters) but the T&E patients required more injections (7.8 *vs*. 5.2). The PRN group included more occult lesions but a subgroup analysis showed that this did not affect outcomes. Surprisingly, the mean numbers of clinic visits were the same in both groups (8.5 *vs*. 8.8). The authors speculated that the poor VA results in the PRN group may have been due to an insufficient number of clinic visits (8.8 compared to a plan of 12) [71].

The best data regarding T&E comes from the national (Norwegian) LUCAS trial [72] that randomized patients to receive ranibizumab or bevacizumab. The average improvements in VA at 1 year were the same for patients receiving ranibizumab and bevacizumab (+8.2 *vs*. +8.0 letters) but the number of injections favored ranibizumab (8.0 *vs.* 8.8; *p* = 0.002). Ranibizumab treated eyes had slightly drier maculas (CST: 249 µm *vs.* 261 µm). Rates of serious systemic adverse events were similar in the two arms but a trend toward more thromboembolic events in the ranibizumab group (4.6% *vs*. 1.4%; *p* = 0.053) was seen. The ranibizumab group experienced a higher rate of procedural related complications [72].

T&E therapy has the least supporting data but it has been adopted by the majority of AMD-treating physicians. The 1-year LUCAS results showed that T&E produces VA gains comparable to those achievable in monthly trials but LUCAS contained no monthly control arms. There is little prospective T&E trial data and none from trials comparing T&E directly with monthly therapy, so comparisons between T&E and other regimens must be made with caution. Additional nationally sponsored T&E trials are not likely to be performed but we hope to see results from smaller prospective T&E trials. T&E decreases the frequency of office visits compared to both PRN and monthly therapy, and requires an injection frequency intermediate between monthly and PRN therapy, while minimizing disease reactivation. With these attributes, the popularity of T&E will likely continue.

Individualized therapy aims to decrease treatment frequency without compromising outcomes but some patients fail to respond adequately to monthly injections. Many of these patients develop favorable anatomic and visual responses for 2 to 3 weeks but worsen by 4 weeks. If these patients are examined and treated monthly, they are characterized as non-responders, but if exams are performed every 2 weeks they would more accurately be called brief responders. These eyes often respond to a series of bevacizumab or alternating bevacizumab/ranibizumab injections administered at 2-week intervals [73]. Once the disease activity is controlled, the treatment intervals can often be extended beyond 4 weeks.

The top-line results from selected important neovascular AMD trials that evaluated monthly and individualized therapies are summarized in Table 1 and Table 2.

### 3.5. Financial

Current costs of anti-VEGF therapy are considerable and reports from the United States and Australia predict significant increases in the total direct costs due to aging of the populations [74,75,76,77]. In 2013, the combined sales of bevacizumab, ranibizumab, and aflibercept were $13.19 billion (US), of which 46.3% was for ophthalmic indications. Ranibizumab accounted for 10% of 2010 Medicare Part B drug sales.

**Table 1:** Phase III registration trials supporting the use of anti-VEGF drugs in eyes with neovascular age-related macular degeneration are listed. Important design characteristics and top-end results are included. peg = pegaptanib; VA = visual acuity; CNVM = choroidal neovascularization; PDT = photodynamic therapy; wk = week.

Drugs and Trials	Trial Design	Important Findings
Pegaptanib (Macugen^®^)	1:1:1	• Pegaptanib treated eyes
		= fewer 15 letter loss
		= better VA
		• Pegaptanib eyes lost a
		mean of −8 letters
VISION	observation: peg 0.3:	• Pegaptanib 0.3 mg q6wk
	peg 3.0	approved
Ranibizumab (Lucentis^®^) ANCHOR (classic CNVM) MARINA (occult CNVM)	1:1:1 PDT: ran 0.3: ran 0.5 1:1:1 observation: ran 0.3: ran 0.5	• Ranibizumab treated eyes = mean gain of +11.3 letters = 95% lost <15 letters
		• Ranibizumab treated eyes = mean gain of +7.4 letters = 95% lost <15 letters
		• Ranibizumab 0.5 mg monthly approved
Aflibercept (Eylea^®^) VIEW 1 and 2	1:1:1:1 ranibizumab q4wk aflibercept 0.5 mg q4wk aflibercept 2 mg q4wk aflibercept 2 mg q8wk	• Approximately 95% in each group lost <15 letters • 1-year gains ranged from +8.2 to +9.4 letters • Aflibercept 2 mg q8wk approved

**Table 2:** Important trials supporting individualized therapy—as-needed and treat and extend—for the treatment of neovascular age-related macular degeneration are listed. Important design characteristics and top-end results are included. BCVA = best corrected visual acuity; CNVM = choroidal neovascularization; PDT = photodynamic therapy; wk = week; PRN = as needed.

Drugs and Trials	Important Findings		
**PRN Trials**			
Ranibizumab (Lucentis^®^) PrONTO SAILOR EXTEND-I SUSTAIN HORIZON	• Mean 1-year BCVA improvement: +9.3 letters • 35% improved by at least 3 lines • Average number of injections over 2 years: 9.9 • BCVA decreased from +5.8 and +7.0 letters (after 3 monthly injections) to +0.5 and +2.3 letters (1 year) • 19.2% of patients improved by at least 15 letters • Average BCVA at 1 year: +9.5 and +10.5 letters • 31% and 37% improved by 15 letters • Intravitreal t_½_ of 6.5 to 7.85 days • Monthly injections × 3 then PRN • Average 12-month BCVA: +3.6 letters • Average injections in final 9 months: 2.7 • Continuation of patients treated in MARINA, ANCHOR & FOCUS • Average injections at 1, 2, 3 years: 2.2, 4.2, 4.3		
Ranibizumab and Bevacizumab (Avastin^®^) (Subramanian *et al*) CATT IVAN MANTA GEFAL	• Average 6-monthV A improvements: Bevacizumab: +14.8 letters Ranibizumab: +7.0 letters (*p* = 0.09) • 12-month BCVA: Bevacizumab monthly: +8.0 letters Ranibizumab monthly: +8.5 letters Bevacizumab PRN: +5.9 letters Ranibizumab PRN: +6.8 letters • Average PRN injections: bevacizumab (7.77), ranibizumab (6.9) • 12-month BCVA: bevacizumab − ranibizumab = −1.99 letters • 12-month BCVA: discontinuous − continuous = −0.35 letters • 12-month BCVA: Bevacizumab (+4.9 letters) Ranibizumab (+4.1 letters) • 12-month BCVA: Bevacizumab (+5.4 letters) Ranibizumab (+3.6 letters)		
**Treat and Extend Trials**			
Engelbert *et al.* Gupta *et al.* LUCAS		• Average LogMAR BCVA improved by 0.01 at 24 months • Average BCVA improved from 20/135 to 20/83 at 2 years • Mean numbers of injections were 8.36 (1 year) and 7.45 (2 years) • 12-month BCVA: Bevacizumab (+8.0 letters) Ranibizumab (+8.2 letters)	

A computerized model evaluated the 5-year costs and utility of current AMD treatments and blindness and compared these figures to BSC (best standard care) and treatment with PDT and pegaptanib [78]. Each treatment show an increased utility compared to BSC if the better-seeing eye is affected by neovascular AMD. The PIER regimen (quarterly after 3 monthly loading doses) administered to the better seeing eye gave the best cost per quality adjusted life-year (QALY) gained at $626,938 (ANCHOR was also evaluated). For bevacizumab, QALY gained is $104,748, though this calculation was based on only 1 year of available clinical data. According to this model, treatment of the worse eye makes sense only when the VA difference between the eyes is less than 9 letters. The costs incurred by blindness increase for treatments that are not able to stabilize vision for at least 5 years. For periods of less than 5 years, the cost of BSC is greater than for all treatments except monthly ranibizumab [78]. BSC costs are high because patients with AMD have increased incidences of anxiety, depression and falls, and increased need for support from social networks [79]. This analysis did not take into account treatment of the worse eye if the better eye suffered severe vision loss.

In CATT, the average yearly drug costs were $23,400 (ranibizumab monthly), $13,800 (ranibizumab PRN), $595 (bevacizumab monthly), and $385 (bevacizumab PRN). The estimated 2-year per patient drug cost varied from $706 for patients receiving bevacizumab as-needed to $44,800 for patients receiving ranibizumab monthly. In the first year of IVAN, continuous and discontinuous charges for eyes treated with ranibizumab were £9656 and £6398, and for those treated with bevacizumab were £1654 and £1509.

The average direct annual medical cost in a treat-and-extend regimen was $16,114, compared to $15,880 for PrONTO and $28,314 for MARINA/ANCHOR. Compared to PrONTO, the first year of T&E requires a fewer office visits and ancillary tests, but more injections [70].

### 3.6. Future Considerations

Individualized therapies for neovascular AMD are used frequently but the treatment burden remains high. To better manage available resources and to limit costs and complications, physicians have devised additional strategies to provide more efficient care. Many offices have created “injection clinics” for patients whose short-term course of intravitreal therapy has already been determined. These clinics enable high patient throughput by limiting the frequency of cognitive evaluations and ancillary tests, thereby allowing physicians to spend more time in the clinic or operating room. Some injection clinics in the United Kingdom use trained nurses to administer intravitreal injections. Preliminary reports suggest that this delivery model is effective and safe with high levels of patient satisfaction, but this strategy has not been studied prospectively and detailed clinical results have not been reported [80,81].

Individualized therapy based upon genetics has become standard-of-care for some oncologic conditions such as breast cancer. The results of each patient’s HER2, Erb2, and BRCA testing determine the chemotherapeutic regimens and surgical approaches. Single nucleotide polymorphisms (SNPs) from several genes—CFH, C2, C3, AMRS, HTRA1, hepatic lipase, VEGF, and VEGFR—are suspected to play a role in the development of advanced AMD but clinical studies cannot consistently predict patients’ responses to therapy based on genetic abnormalities [82,83,84,85]. Nonetheless, genetic studies continue with the hope that individualized therapy can someday be tailored to the presence or absence of AMD susceptibility genes.

Physicians continue to use thermal laser photocoagulation, pegaptanib, corticosteroids, and photodynamic therapy for selected cases of neovascular AMD, but most patients are treated with intravitreal injections of bevacizumab, ranibizumab or aflibercept. Level I evidence from randomized, controlled AMD trials suggests that +10 letters is the therapeutic limit achievable with anti-VEGF monotherapy. Specific and non-specific molecular therapies that target platelet derived growth factor, complement factors, and chemokine receptors are being tested in combination with anti-VEGF therapy, in hopes of achieving an additional effect. These drugs are currently in various stages of development—from pre-clinical to phase III trials—though none is expected to receive FDA approval within the next 3 years.

Patient compliance with anti-VEGF therapy remains a challenge despite the decreased frequency of intravitreal injections and office visits afforded by individualized therapy. New routes of drug administration, including extended release implants, encapsulated cell technology, topical formulations, and oral pills, are being developed. Successful development of these drugs and devices will enable physicians to improve visual outcomes with combination therapy, and choose the best method of drug administration for each patient. As the number of treatment options increases, so will the importance of careful counselling with each patient.

## 4. Summary

Monthly injections may not be appropriate for many patients because of the associated “physical and psychological burdens” [53], and the perceived increased risks of AEs. As-needed (PRN) treatment produces good anatomic and visual results, but maculas generally remain thicker than with monthly therapy and mean VA improvements lag by 1–3 letters at one year. Up to 50% fewer injections are given during the first year but patients still require monthly assessments. Treat-and-extend may improve VA by +8 letters at one year but randomized head-to-head trials against monthly therapy need to be performed.

## 5. Conclusions

Individualized therapy is a valuable treatment strategy that is used for most patients with neovascular AMD. Despite years of trials using both monthly and individualized therapy, the optimal treatment strategy has not yet been defined and treatment decisions should be based on an in-depth discussion between the physician and patient.
